# Navigating global collaboration: challenges faced by the international network on esophageal atresia

**DOI:** 10.1186/s13023-024-03250-6

**Published:** 2024-08-21

**Authors:** Frédéric Gottrand, Usha Krishnan, Anke Widenmann, Michaela Dellenmark Blom, Luigi Dall’Oglio, Rene Wijnen, Michiel van Wijk, JoAnne Fruithof, Daniel von Allmen, Tom Kovesi, Christophe Faure

**Affiliations:** 1 Reference Center for Congenital Oesophageal Anomalies, Univ. Lille, CHU Lille, Infinite U1286, Lille, F-59000 France; 2 Discipline of Pediatrics, School of Women’s and Children’s Health, Faculty of Medicine, University of New South Wales, Randwick, NSW Australia; 3 EAT, Federation of esophageal atresia and trachea-esophageal fistula, Stuttgart, Germany; 4 Department of Pediatrics, University of Gothenburg Institute of Clinical Sciences, Gothenburg, Sweden; 5 Digestive Endoscopy and Surgery Unit, Bambino Gesù Children’s Hospital, IRCCS Rome, Rome, Italy; 6 Department of Pediatric Surgery and Intensive Care Children, Erasmus MC Sophia Children’s Hospital, Wytemaweg 80, Rotterdam, 3015 CD The Netherlands; 7 Department of Gastroenterology, Amsterdam UMC Location AMC, Amsterdam, North Holland The Netherlands; 8 Department of Pediatric Surgery and Surgical Services, Cincinnati Children’s Hospital Medical Center, Cincinnati, OH USA; 9 Children’s Hospital of Eastern Ontario, University of Ottawa, Ottawa, Canada; 10 Department of Pediatrics, CHU Ste-Justine Research Centre, Université de Montréal, Montréal, QC H3T 1C5 Canada

**Keywords:** Gastroenterology, Esophageal atresia, Long life disease; transition to adult medicine

## Abstract

The International Network on Esophageal Atresia (INoEA) stands as a beacon of collaboration in addressing the complexities of this congenital condition on a global scale. The eleven board members, from various countries (USA, Canada, France, Australia, Italy, Sweden, Germany, and The Netherlands) and backgrounds (pediatric gastroenterology, pediatric surgery, pediatric pulmonology, nursing, and parents) met in a face-to-face symposium in Lille in November 2023, to identify challenges and solutions for improving global collaboration of the network.

The International Network on Esophageal Atresia (INoEA) stands as a beacon of collaboration in addressing the complexities of this congenital condition on a global scale. INoEA brings together professionals from diverse healthcare systems and specialties, each with its unique challenges and strengths, as well as patients and family support groups. INoEA was created in 2010, to support the organization of regular International meetings on EA every 2 to 3 years, promote international research efforts in the field of esophageal atresia (EA), facilitate development of multicenter studies and collaboration between centers, and develop consensus statements [1–4] and clinical guidelines for the optimal management of this complex condition and thereby improve patient outcomes and quality of life. So far INoEA has collaborative connections with European societies/groups (The European Reference Network on Rare Inherited and Congenital Anomalies (ERNICA), The European Society for Paediatric Gastroenterology Hepatology and Nutrition (ESPGHAN)), North American societies (North American Society For Pediatric Gastroenterology, Hepatology & Nutrition (NASPGHAN)), and International (The International Society for Diseases of the Esophagus (ISDE), The Aerodigestive society, Patients associations federation (EAT)).

Eleven board members of INoEA from various countries (USA, Canada, France, Australia, Italy, Sweden, Germany, and The Netherlands) and backgrounds (pediatric gastroenterology, pediatric surgery, pediatric pulmonology, nursing, and parents) met in a face-to-face symposium in Lille in Nov 2023, to identify challenges and solutions for improving global collaboration of the network. We have described these below.

## Data sharing and privacy concerns

The collaborative nature of INoEA relies heavily on data sharing among participating institutions. In fact, in rare diseases, such as EA, international data sharing is key to high quality research. However, navigating the complex landscape of data privacy laws and ethical considerations poses a significant challenge. Establishing robust frameworks for secure data sharing that comply with international legal regulations while fostering transparency is crucial. We will explore the feasibility of a global agreement between participating centers fulfilling the international rules of privacy protection. An alternative could be to define a common set of data each member includes in their local database to facilitate future collaborative work for selection of patients for studies.

## Translating recommendation into clinical practice

International guidelines on EA are now endorsed by different scientific societies. In rare diseases, many statements in clinical guidelines are based on expert opinion and therefore recommendations may differ. It was shown that implementation of our guidelines is as yet insufficient [5].

One of the ways to improve implementation of guidelines into practice is to seek endorsement of all scientific societies that are involved worldwide and put them under the same umbrella. It will not only make them more visible and easier accessible for patient associations, but it will also establish a robust starting point for international trials. INoEA will stimulate more intensive collaboration between all scientific societies to reach this goal. To acknowledge the lifelong consequences faced by patients with EA, societies focusing on adult patients will also be asked to collaborate. Adult-oriented professionals will be actively asked to participate in our next INoEA meeting in Istanbul in 2025.

In addition, different channels will be explored to communicate current and future guidelines: apps, social networks, Orphanet [6], Translation into different languages, and editing short or publicized versions would also improve dissemination.

## Building tools together for everyone

A core outcome set (Ocelot study, Ocelot study < TOFS | OA/TOF Support, ongoing ), and disease-specific quality of life questionnaires have been developed recently in EA [7, 8], but still need to be translated and transculturally validated in multiple languages/countries. An international multiphase study to develop patient-reported outcome and experience measurements focusing on symptom burden in people born with EA and their experiences of the received care/treatment has also been established. These are huge ongoing efforts and reflect future contribution from members on the board of INoEA. Patient-reported outcomes measurement development will involve all the disciplines including medical and allied health professionals and parent support groups which are a part of the network.

## Resource disparities

Participating institutions within INoEA may face significant disparities in resources, including funding, technology, and expertise, and there remains a big gap in the quality of care and prognosis between developed and developing countries [9]. Bridging these gaps is essential to ensure that all members can actively contribute to the network’s objectives. Encouraging resource-sharing initiatives, mentorship programs, and capacity-building efforts can create a more equitable environment, empowering all members to actively engage in research and patient care initiatives including training, and e-learning.

## Basic science/translational (induced pluripotent stem cells, tissue engineering, genetics, omics)

Recent advances in research technologies and new approaches may revolutionize care in EA. INoEA will summarize and connect all these initiatives into a review paper on this topic.

## Conclusion

The INoEA is at the forefront of global and collaborative efforts to understand and address the challenges posed by this complex congenital condition. To navigate the complexities of international collaboration successfully, INoEA must address diverse healthcare landscapes, data sharing concerns, communication/dissemination barriers and resource disparities. By overcoming these challenges, the network can harness the collective expertise of its members to drive meaningful advancements in the field of EA research and ultimately improve outcomes for patients worldwide. While medical advancements have significantly improved the outlook for individuals with EA, new challenges necessitate a paradigm shift in our approach. A holistic, patient-centered model that addresses long-term complications, prioritizes mental health, embraces technological innovations, ensures a smooth transition to adult care, and empowers patients and parents is crucial for overcoming the evolving landscape of EA.

## Data Availability

Not applicable.

## Abbreviations

INoEA: International Network on Esophageal Atresia
EA: Esophageal atresia
ERNICA: The European Reference Network on Rare Inherited and Congenital Anomalies
ESPGHAN: The European Society for Paediatric Gastroenterology Hepatology and Nutrition
NASPGHAN: North American societies (North American Society For Pediatric Gastroenterology, Hepatology & Nutrition
ISDE: The International Society for Diseases of the Esophagus
EAT: Patients associations federation
